# Supplementary material for: B cell immunity to the Lassa virus glycoprotein is a correlate of vaccination-induced virus control in mice
Source: Nat Commun. 2026 Apr 6;17:4908. doi: 10.1038/s41467-026-71472-2 (PMC13230540; doi:10.1038/s41467-026-71472-2)
Supplement: Supplementary file 1 — Supplementary information [file 41467_2026_71472_MOESM1_ESM.pdf]

Supplementary information

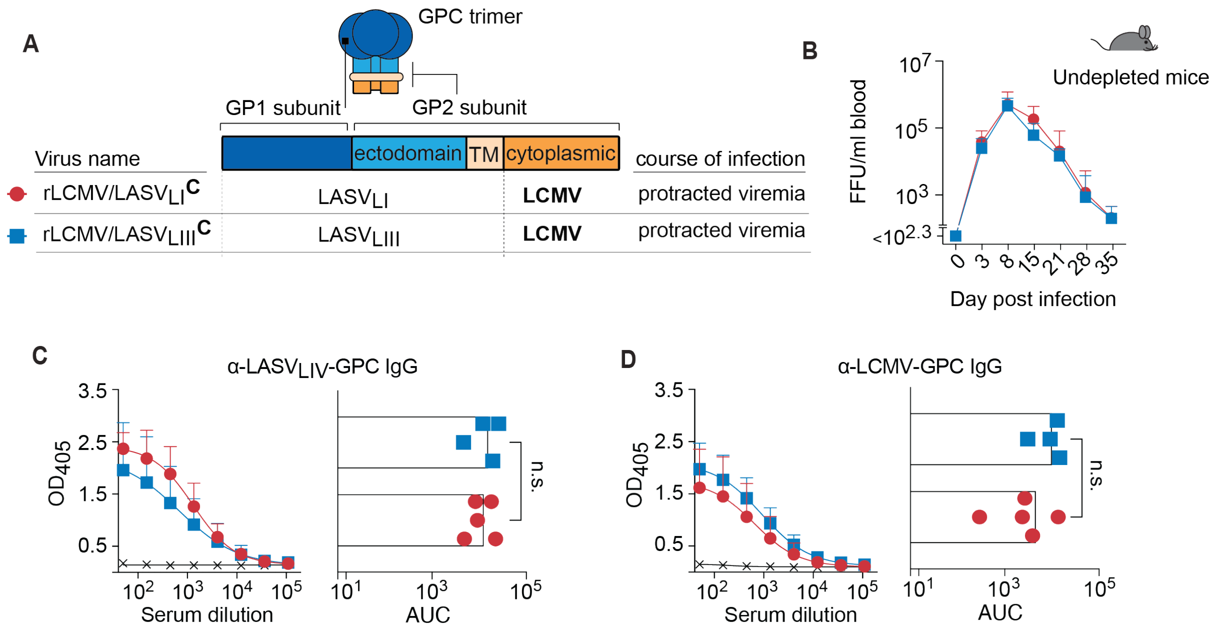

Figure S1. The glycoproteins of LASV LI and LIII elicit cross-reactive antibody responses to the glycoproteins of LASV LIV and LCMV. (A) Table listing the remaining recombinant LCMV viruses used, the origin of each GPC domain they express and the course of infection they elicit in mice (B) WT mice were infected with the designated viruses and viremia was measured over time. (C,D) Serum IgG titers against $LASV_{LIV}$-GPC (C) and against LCMV-GPC (D) at d35 of the experiment in (B). ELISA curves and their area under the curve (AUC) are shown with X representing blank. Symbols on curves represent the mean+SD of n=4 ($rLCMV/LASV_{LIII}{}^{C}$) and n=5 ($rLCMV/LASV_{LI}{}^{C}$) mice, symbols on AUC bars of (C,D) represent the same individual mice. One representative experiment out of two similar ones is shown in (B-D). *: $p \leq 0.05$; **: $p \leq 0.01$; n.s.: $p > 0.05$ by 1-way ANOVA with Bonferroni's post-test.

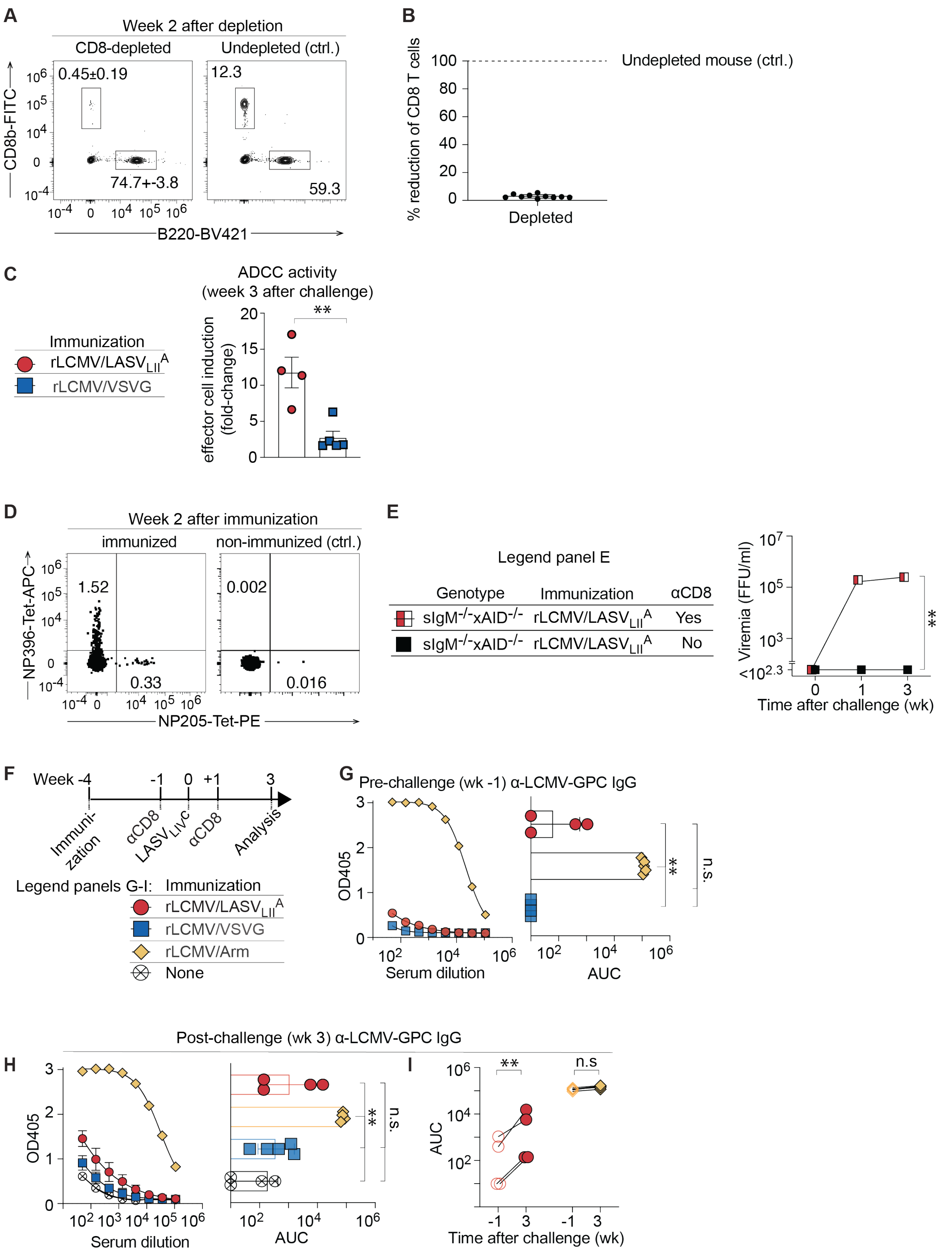

Figure S2. Antibody cross-reactivity between LASV GPCs of different lineages suppresses viremia

(A,B) CD8 T cell analysis after depletion. Illustrative FACS plots of CD8 T cell-depleted and non-depleted control mice 2 weeks after antibody injection. (B) Relative abundance of CD8 T cells as

compared to non-depleted WT mice. Each symbol in (B) represents an individual animal. Bars indicate the mean±SD. One out of two similar experiments is shown.

(C) We tested sera of WT mice immunized with rLCMV/$LASV_{LII}^{A}$ (n=4) or rLCMV/VSVG (n=5) (experiment reported in Fig. 2A-E) in an ADCC surrogate assay, determining their ability to trigger FcγRIV-mediated effector cell activation in the presence of target cells expressing LASV-GPC. Luminescence values are presented as fold induction relative to technical background. Each symbol in (C) represents an individual animal. Bars indicate the mean±SD.

(D) WT mice were immunized with rLCMV/$LASV_{LII}^{A}$ and two week 2 later we determined virus-specific CD8 T cells specific for the immunodominant nucleoprotein-derived epitopes NP396 and NP205 in blood using MHC class I tetramers. Illustrative FACS plots from (n=5) rLCMV/$LASV_{LII}^{A}$-immunized and (n=2) non-immunized control mice analyzed in one experiment are shown. Values in quadrants indicate the mean percentage of gated cells.

(E) $sIgM^{-/-}AID^{-/-}$ mice (n=10) were depleted of CD8 T cells at wk -5 or left undepleted (n=2), immunized with rLCMV/$LASV_{LII}^{A}$ at wk -4, again depleted of CD8 T cells or left undepleted at wk -1 and challenged with rLCMV/$LASV_{LIV}^{C}$ at wk 0. rLCMV/$LASV_{LIV}^{C}$ viremia was monitored over time. Symbols show the mean of n=10 CD8-depleted and n=2 non-depleted mice. Data from CD8-depleted $sIgM^{-/-}AID^{-/-}$ mice are combined from two experiments and are the same as those shown in Fig. 2F, non-depleted mice are from one experiment.

(F) Experimental outline for panels (G-I). WT mice were immunized with either rLCMV/$LASV_{LII}^{A}$ (n=4), rLCMV/VSVG (n=5), rLCMV/Arm (n=5), or none (n=4) at wk -4, depleted of CD8 T cells at wk -1, challenged with rLCMV/$LASV_{LIV}^{C}$ at wk 0, and depletion of CD8 T cells was repeated at wk +1. (G) Anti-LCMV-GPC IgG titers at wk -1. (H) Anti-LCMV-GPC IgG and (I) matched proportional increase of anti-LCMV-GPC IgG AUC from wk -1 to wk 3. Each symbol in (G right, H right, I) represents an individual animal. Bars indicate the mean±SD. Symbols in (G left, H left) show the mean±SD. Panels (G-I) report one out of two similar experiments. Unpaired two-tailed Student's t-test was used in (C), one-way ANOVA with Tukey's post-test in (G,H), two-way ANOVA with Sidak's post-test was performed to assess significance between pre- and post-challenge (I). **: $p \leq 0.01$; *: $p \leq 0.05$; n.s.: $p > 0.05$.

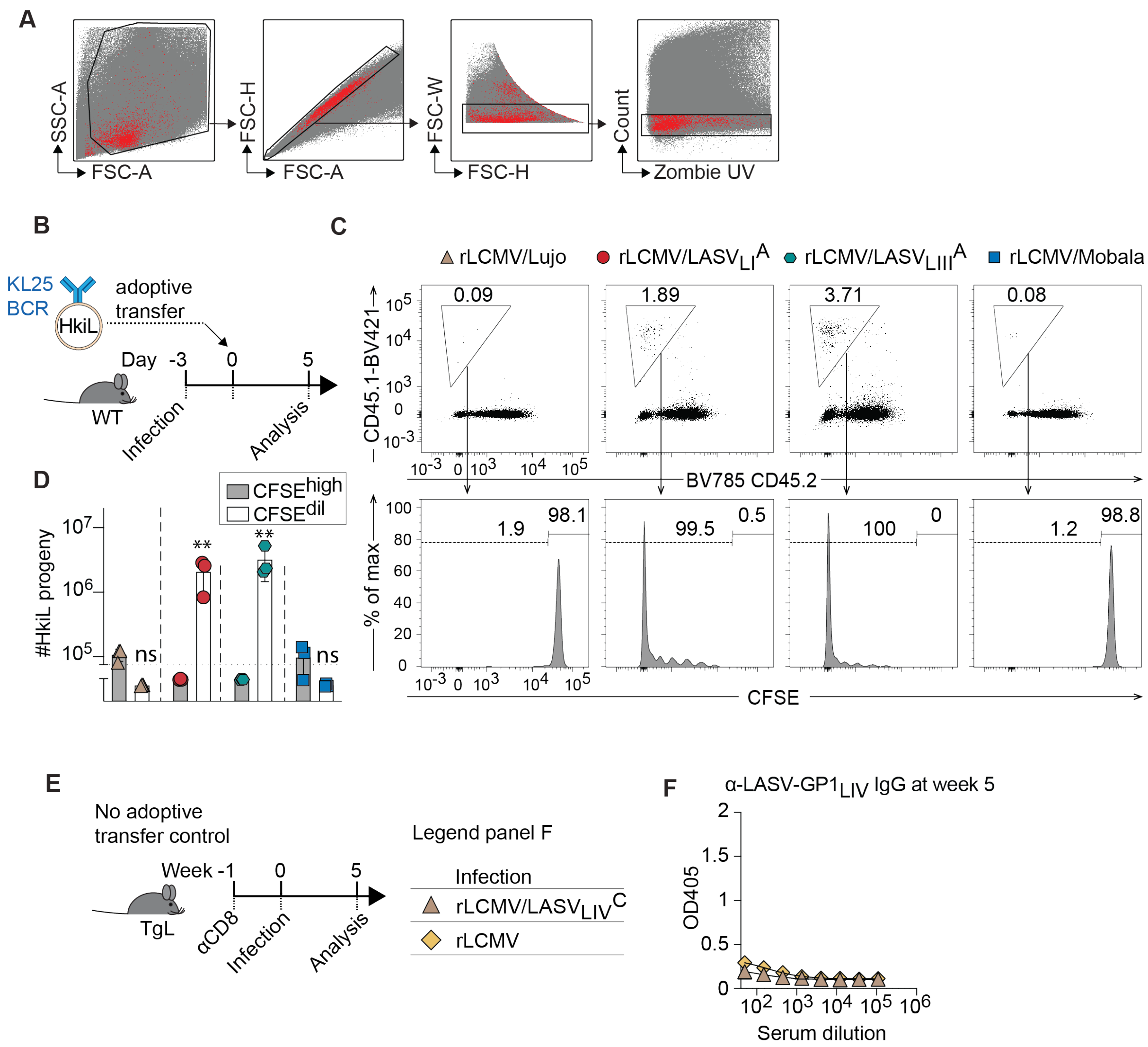

Figure S3. LCMV-GP1-specific B cells proliferate and affinity mature in response to LASV-GPC

(A) General gating strategy for lymphocyte analysis. (B) Experimental outline for (C-D) WT mice were infected at d-3 with either rLCMV/LASV$_{LI}^{A}$ (n=3), rLCMV/LASV$_{LIII}^{A}$ (n=3), rLCMV/Mobala (n=3) or rLCMV/Lujo (n=3) at d-3, and received 10^6 CFSE-labeled HkiL splenocytes by adoptive transfer at d0, followed by flow cytometric analysis on d5. (C) Gating of adoptive transferred HkiL cells in spleen (CD45.1^{+}, top row) and determination of CFSE dilution (bottom row). Representative (n=3) FACS plots are shown, numbers indicate the percentage of gated cells. (D) Total splenic count of non-proliferated (CFSE^{high}) HkiL cells and of proliferated HkiL cells with diluted CFSE levels (CFSE^{dil}). Symbols in (D) show individual mice.

(E) TgL mice were depleted of CD8 cells at week -1, infected with either rLCMV (n=2) or rLCMV/LASV$_{LIV}^{C}$ (n=6) at day (-1) followed by analysis at wk 5. (F) Anti-LASV$_{LIV}$-GP1 IgG by ELISA. Symbols in (F) show the mean of n=2 rLCMV-infected (from one experiment) and n=6 rLCMV/LASV$_{LIV}^{C}$-infected mice (combined from two experiments). 1-way ANOVA with Dunnett's post-test was performed

for (D) comparing the percentage of $CFSE^{dil}$ cells to those of rLCMV/VSVG-immunized animals in Fig. 3F, which were analyzed in the same experiment. **: $p \leq 0.01$; *: $p \leq 0.05$; n.s.: $p > 0.05$.

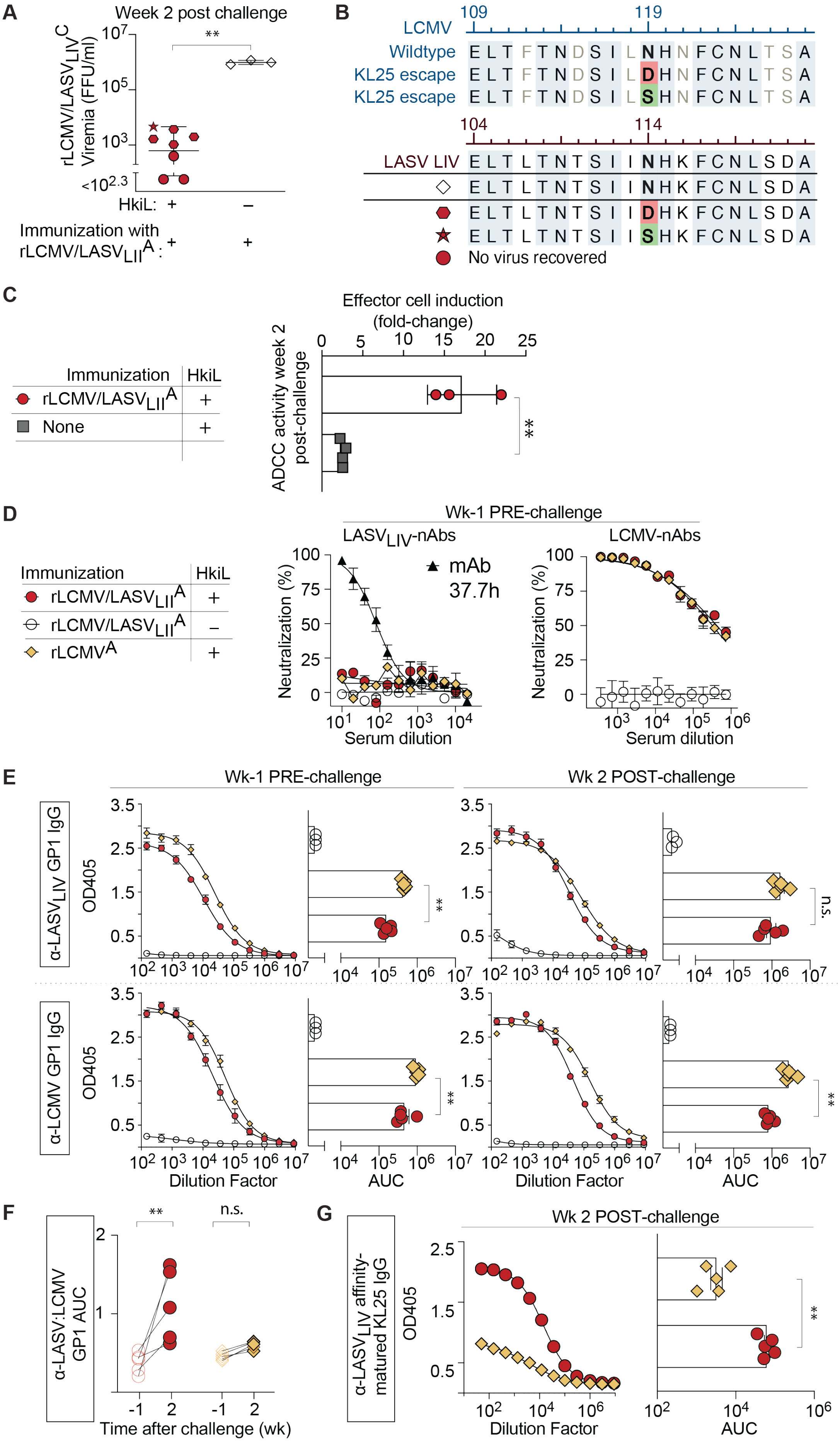

A
Week 2 post challenge
rLCMV/LASVLIVC Viremia (FFU/ml)
HkiL: + –
Immunization with rLCMV/LASVLIIA: + +
B
LCMV
Wildtype
KL25 escape
KL25 escape
LASV LIV
No virus recovered
C
Immunization HkiL
rLCMV/LASVLIIA +
None +
Effector cell induction (fold-change)
ADCC activity week 2 post-challenge
D
Wk-1 PRE-challenge
LASVLIV-nAbs
LCMV-nAbs
Immunization HkiL
rLCMV/LASVLIIA +
rLCMV/LASVLIIA –
rLCMVA +
mAb 37.7h
Neutralization (%)
Serum dilution
E
Wk-1 PRE-challenge
Wk 2 POST-challenge
α-LASVLIV GP1 IgG
α-LCMV GP1 IgG
OD405
Dilution Factor
AUC
n.s.
F
α-LASV:LCMV GP1 AUC
Time after challenge (wk)
n.s.
G
Wk 2 POST-challenge
α-LASVLIV affinity-matured KL25 IgG
OD405
Dilution Factor
AUC

Figure S4. LASV-GPC but not LCMV-GPC priming enables control of rLCMV/LASV$_{LIV}^{C}$ viremia by HkiL cells.

(A,B) In the experiments conducted as outlined in Fig. 4A,F, TgL mice were depleted of CD8 T cells at week -5. At wk-4 they were immunized with rLCMV/LASVL$_{II}^{A}$ and given 10^4 HkiL cells (n=8) or none (n=3) as indicated in the chart. CD8 T cell depletion was repeated at wk-1, and at wk0 the animals were challenged with rLCMV/LASV$_{LIV}^{C}$. (A) rLCMV/LASV$_{LIV}^{C}$ viremia at wk2 is shown for select animals from the experiments reported in Figs. 4B,G. (B) Virus was grown from the blood of individual mice and the viral glycoprotein sequence was determined. LASV-GPC amino acids 104-123 are displayed (bottom), aligned to the homologous LCMV-GPC sequence stretch (Aa 109-128; top), together with known KL25 escape mutations[70] in LCMV-GPC. From the animals with undetectable or very low-level viremia, virus outgrowth failed (no virus recovered). Symbol types and colors in panel (A; each symbol representing an individual mouse) refer to the amino acid sequence as determined in (B), revealing that rLCMV/LASV$_{LIV}^{C}$, when persisting in HkiL recipients at low levels, had acquired typical KL25 escape mutations, whereas rLCMV/LASV$_{LIV}^{C}$ in mice without HkiL cell transfer was devoid of such mutations. Each symbol represents one mouse.

(C) CD8 T cell-depleted TgL recipients of HkiL cells were vaccinated with rLCMV/LASV$_{LII}^{A}$ (n=3) or left unvaccinated (n=4) and challenged with rLCMV/LASV$_{LIV}^{C}$ as outlined in Fig. 4A. Sera collected two weeks after challenge (from one out of the two experiment that were combined for Fig. 4B-E) were tested in an ADCC surrogate assay, determining their ability to trigger FcγRIV-mediated effector cell activation in the presence of target cells expressing LASV-GPC. Luminescence values are presented as fold induction relative to technical background. Each symbol represents an individual animal, bars indicate the mean±SD.

(D-G) Data pertaining to the experiment in Fig. 4F. (D) LASV$_{LIV}$- and LCMV-neutralizing antibody (nAb) responses at wk-1 before challenge. (E) ELISA measurement anti-LASV$_{LIV}$-GP1 IgG titers and anti-LCMV-GP1 titers at wk -1 and wk 2 respective to challenge. (F) Ratio of the AUC of anti-LASV$_{LIV}$-GP1 IgG to anti-LCMV-GP1 before and after challenge. (G) Competition ELISA for relative affinity determination (KL25 epitope binning) at wk2 post challenge. Total anti-LASV$_{LIV}$-GP1 IgG binding to previously blocked LASV$_{LIV}$-GP1 by KL25. Horizontal bars denotate the mean±SD with symbols representing individual mice. In (D-F) one out of two independent experiments with n=5 (HkiL+rLCMV/LASV$_{LII}^{A}$ and HkiL+rLCMV^{A}) or n=3 (rLCMV/LASV$_{LII}^{A}$-only) mice per group. Panel (G) shows one out of two similar experiments. Two-way ANOVA with Sidak's post-test was conducted in (F) to assess differences between pre- and post-challenge, unpaired two-tailed Student's *t*-test was used in (A,C,E,G). **: $p \leq 0.01$; *: $p \leq 0.05$; n.s.: $p > 0.05$.

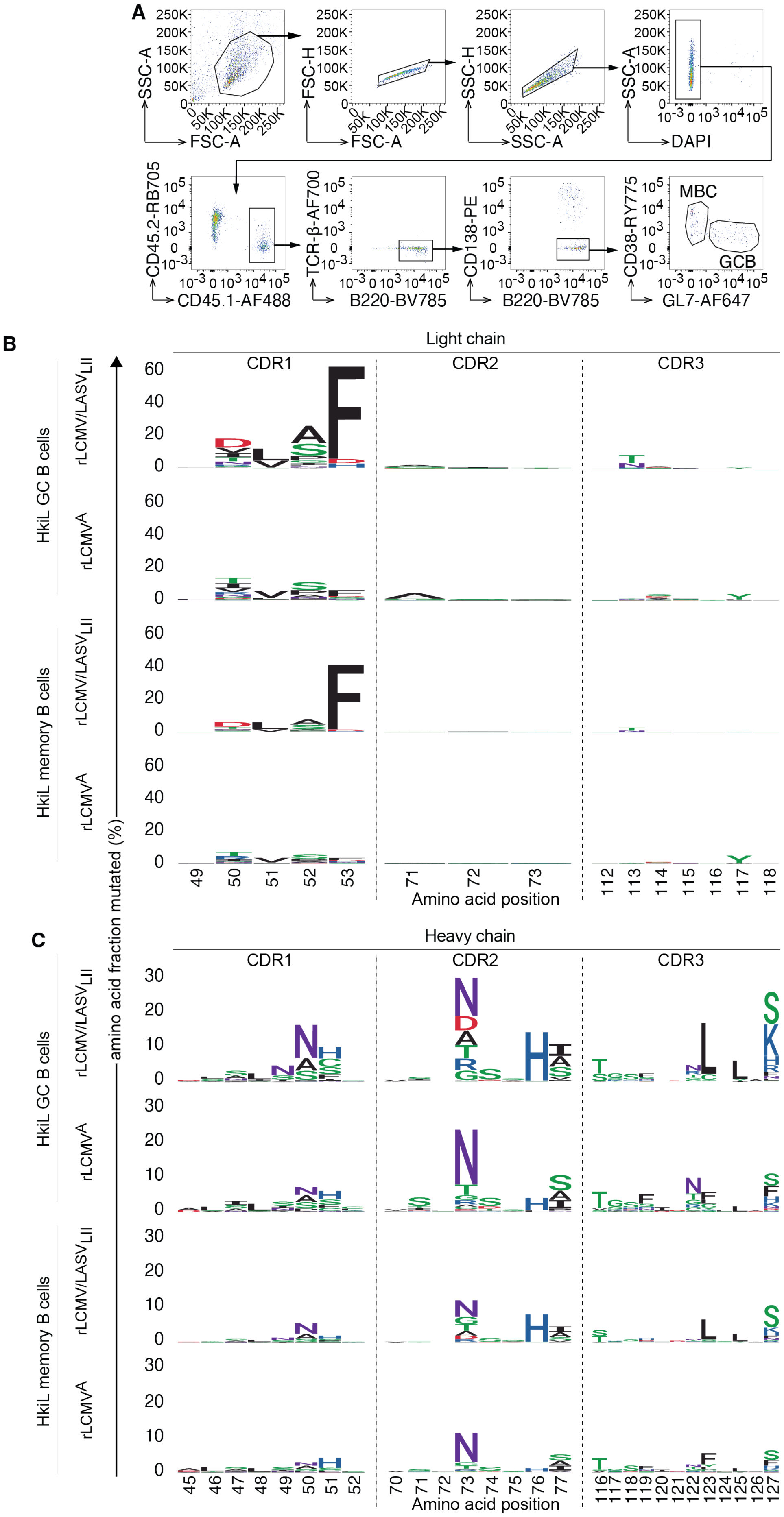
A
SSC-A
FSC-A
FSC-H
SSC-H
DAPI
CD45.2-RB705
CD45.1-AF488
TCR-β-AF700
B220-BV785
CD138-PE
CD38-RY775
GL7-AF647
MBC
GCB
B
Light chain
CDR1
CDR2
CDR3
HkiL GC B cells
HkiL memory B cells
rLCMV/LASV
rLCMVA
amino acid fraction mutated (%)
Amino acid position
C
Heavy chain

Figure S5. The immunizing GPC determines the extent of HkiL cell hypermutation and clonal diversification.

(A) Gating strategy for the sorting and scRNAseq-based V(D)J determination of HkiL GC B cells and MBCs in the experiment to Fig. 5 and Fig. S5B,C.

(B,C) Recurrent mutations in the complementarity-determining regions (CDRs) of the light chain (B) and heavy chain (C) of HkiL GC B cells and MBCs as determined in the scRNAseq experiment to Fig. 5. Cumulated data from n=4 (rLCMV^{A}) and n=5 (rLCMV/LASV$_{LII}$^{A}) mice per group are shown.

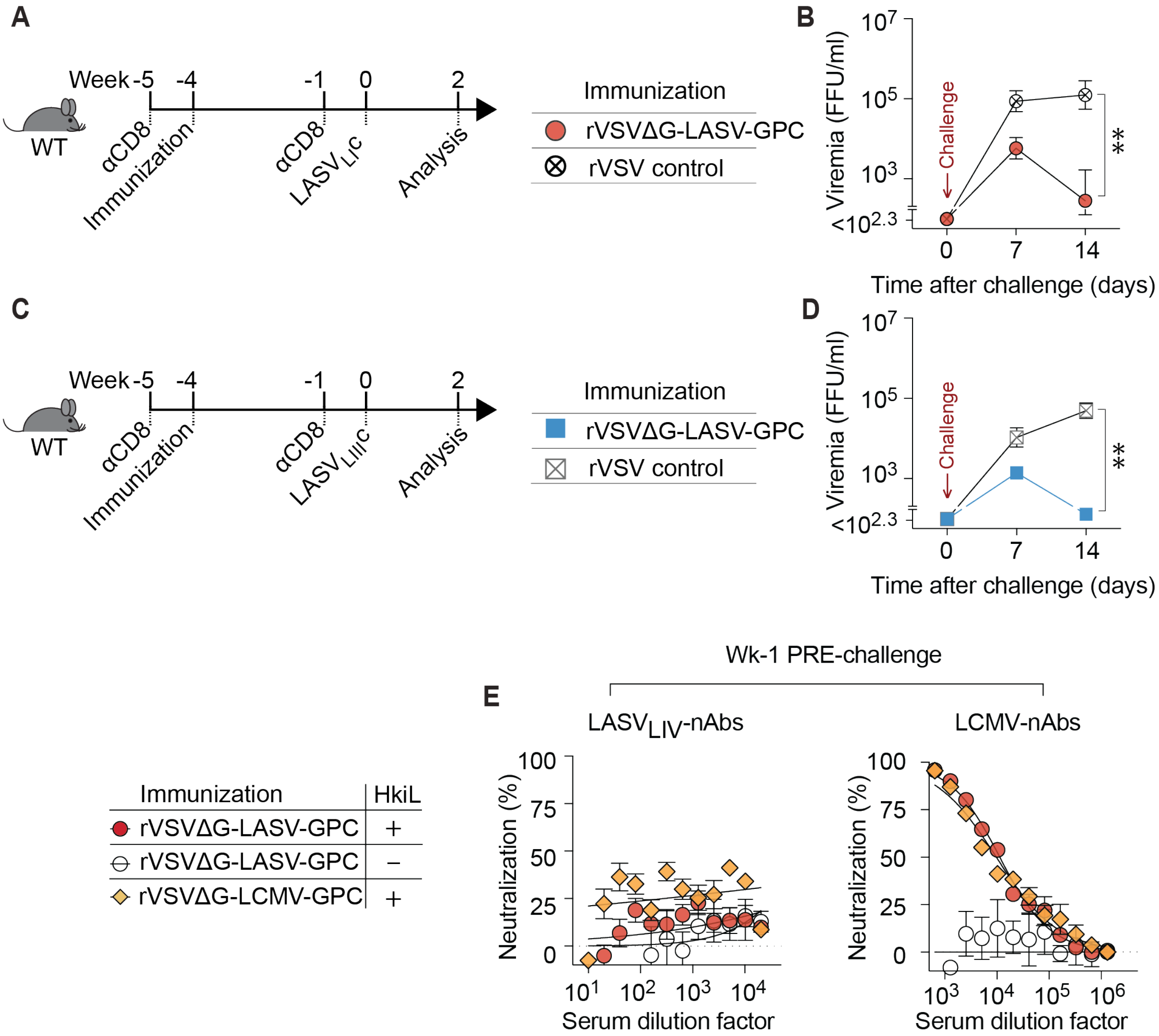

Figure S6. A clinical-stage Lassa vaccine candidate suppresses viremia independently of CD8 T cells and drives B cell affinity maturation.

(A) Outline of the experiment in panel (B). At wk-5, WT mice were depleted of CD8 T cells and at wk-4 they were immunized as indicated with either rVSVΔG-LASV-GPC (n=5) or with a negative control vector expressing the irrelevant Zaire Ebolavirus glycoprotein (VSVΔG/ZEBOVGP[98]; rVSV control) (n=4). CD8 T cell depletion was repeated at wk-1, and at wk0 the animals were challenged with rLCMV/$LASV_{LI}C$. (B) Viremia was monitored over time.

(C) Outline of the experiment in panel (D). At wk-5, WT mice were depleted of CD8 T cells and at wk-4 they were immunized as indicated with either rVSVΔG-LASV-GPC (n=4) or with a negative control vector expressing the irrelevant Zaire Ebolavirus glycoprotein (rVSV control) (n=4). CD8 T cell depletion was repeated at wk-1, and at wk0 the animals were challenged with rLCMV/$LASV_{LIII}C$. (B) Viremia was monitored over time.

(E) $LASV_{LIV}$- and LCMV-nAb responses at wk-1 of the experiment reported in figure 7F-M. n=6 (HkiL+rVSVΔG-LASV-GPC), n=4 (HkiL+rVSVΔG-LCMV-GPC), n=3 (rVSVΔG-LASV-GPC-only) mice

per group. Symbols in (B,D) represent the mean±SEM. Two-way ANOVA was conducted for statistical analysis in (B,D). **: $p \leq 0.01$.s

Supplementary Table I. Antibody conjugates used in flow cytometry

Antigen	Fluorophore	Dilution	Clone	Provider	Catalog number
B220	BV785	1:800	RA3-6B2	Biolegend	103246
B220	BV421	1:800	RA3-6B2	Biolegend	103251
Bcl6	BUV615	1:400	K112-91	BDBiosciences	568061
IRF4	RB744	1:600	Q9-343	BDBiosciences	Custom
CD19	BUV496	1:200	1D3	Thermofisher	364-0193-82
CD19	PE	1:200	6D5	Biolegend	115508
CD19	FITC	1:100	1D3	Biolegend	152404
CD138	PE	1:200	REA104	Miltenyi	130-120-810
CD138	APC	1:200	281-2	Biolegend	142506
CD138	BV605	1:400	281-2	Biolegend	142531
CD138	BV421	1:400	281-2	Biolegend	142523
GL7	AF647	1:1000	GL7	Biolegend	144606
GL7	PacBlue	1:800	GL7	Biolegend	144614
CD38	AF488	1:400	90	Biolegend	102714
CD38	BUV395	1:200	90	BDBiosciences	740245
CD38	PE-vio770	1:400	REA616	Miltenyi	130-125-522
CD38	BV480	1:200	90	Thermofisher	414-0381-82
CD45.1	AF488	1:400	A20	Biolegend	110717
CD45.1	SUV387	1:200	A20	Biolegend	110761
CD45.1	BV421	1:200	A20	Biolegend	110732
CD45.2	SB436	1:200	104	Thermofisher	62-0454-82
CD45.2	BUV661	1:200	104	Thermofisher	376-0454-82
CD45.2	RB705	1:200	104	BDBiosciences	757503
CD45.2	BV785	1:200	104	Biolegend	109839
IgD	APC-cy7	1:200	11-26c.2a	Biolegend	405715
IgD	Percp-vio700	1:200	REA772	Miltenyi	130-111-500
IgM	Percp-efluor710	1:200	II/41	Thermofisher	46-5790-80
IgM	Viogreen	1:100	REA979	Miltenyi	130-116-216
TCR-β	AF700	1:200	H57-597	Biolegend	109223
CD8β	FITC	1:200	YTS156.7.7	Biolegend	126605
CD4	R718	1:200	RM4-5	BDBiosciences	566939
CD62L	PE-cy7	1:200	MEL-14	Biolegend	104417
CD44	BUV805	1:600	IM7	BDBiosciences	741921
CD22	BV786	1:1500	Cy34.1	BDBiosciences	740871
