# Supplementary material for: B cell immunity to the Lassa virus glycoprotein is a correlate of vaccination-induced virus control in mice
Source: Nat Commun. 2026 Apr 6;17:4908. doi: 10.1038/s41467-026-71472-2 (PMC13230540; doi:10.1038/s41467-026-71472-2)
Supplement: Supplementary file 2 — Reporting Summary [file 41467_2026_71472_MOESM2_ESM.pdf]

nature portfolio

Corresponding author(s): Tiago Abreu-Mota, Daniel D. Pinschewer

Last updated by author(s): Feb 18, 2026

Reporting Summary

Nature Portfolio wishes to improve the reproducibility of the work that we publish. This form provides structure for consistency and transparency in reporting. For further information on Nature Portfolio policies, see our Editorial Policies and the Editorial Policy Checklist.

Statistics

For all statistical analyses, confirm that the following items are present in the figure legend, table legend, main text, or Methods section.

n/a	Confirmed	
☐	☒	The exact sample size (*n*) for each experimental group/condition, given as a discrete number and unit of measurement
☐	☒	A statement on whether measurements were taken from distinct samples or whether the same sample was measured repeatedly
☐	☒	The statistical test(s) used AND whether they are one- or two-sided<br>*Only common tests should be described solely by name; describe more complex techniques in the Methods section.*
☒	☐	A description of all covariates tested
☐	☒	A description of any assumptions or corrections, such as tests of normality and adjustment for multiple comparisons
☐	☒	A full description of the statistical parameters including central tendency (e.g. means) or other basic estimates (e.g. regression coefficient) AND variation (e.g. standard deviation) or associated estimates of uncertainty (e.g. confidence intervals)
☐	☒	For null hypothesis testing, the test statistic (e.g. *F*, *t*, *r*) with confidence intervals, effect sizes, degrees of freedom and *P* value noted<br>*Give P values as exact values whenever suitable.*
☒	☐	For Bayesian analysis, information on the choice of priors and Markov chain Monte Carlo settings
☒	☐	For hierarchical and complex designs, identification of the appropriate level for tests and full reporting of outcomes
☒	☐	Estimates of effect sizes (e.g. Cohen's *d*, Pearson's *r*), indicating how they were calculated

Our web collection on statistics for biologists contains articles on many of the points above.

Software and code

Policy information about availability of computer code

Data collection	n.a.
Data analysis	The sequencing data were processed with the BD rhapsody workflow (version 2.4b3.post1). All subsequent data analysis was performed in R (version 4.4.2) and Bioconductor (version 3.20) and tidyverse. The V(D)J data (file BCR_VDJ_Dominant_Contigs_AIRR.tsv) was filtered for high quality, productive contigs and complete observation of heavy and light chain per cell (101340 cells). The V(D)J data was combined with the mRNA Immune panel and sample hashtag data using the SingleCellExperiment package. The default sample demultiplexing of the BD workflow was corrected using the emptyDrops function of the DropletUtils package. This gave rise to a final set of 77835 cells that could reliably be assigned to a specific sample. The mRNA Immune panel expression data was used to classify B cells as memory or germinal center (GC) using clustering and gene marker lists. The subsequent clonotype analysis included only B cells of IgG isotype (74015 cells). Amino acid substitution rates were calculated by aligning chains to the respective HkiL reference sequences using pairwiseAlignment and mismatchTable functions of the pwalign package.

For manuscripts utilizing custom algorithms or software that are central to the research but not yet described in published literature, software must be made available to editors and reviewers. We strongly encourage code deposition in a community repository (e.g. GitHub). See the Nature Portfolio guidelines for submitting code & software for further information.

Data

Policy information about availability of data

All manuscripts must include a data availability statement. This statement should provide the following information, where applicable:

- Accession codes, unique identifiers, or web links for publicly available datasets
- A description of any restrictions on data availability
- For clinical datasets or third party data, please ensure that the statement adheres to our policy

Raw data of the experimental results reported in this study will be deposited with Zenodo and made publicly available as of the date of publication under the DOI 10.5281/zenodo.17794274. Single cell RNA sequencing data have been deposited with the National Center for Biotechnology Information Gene Expression Omnibus (GEO) under the accession number GSE313953.

Research involving human participants, their data, or biological material

Policy information about studies with human participants or human data. See also policy information about sex, gender (identity/presentation), and sexual orientation and race, ethnicity and racism.

Reporting on sex and gender	n.a.
Reporting on race, ethnicity, or other socially relevant groupings	n.a.
Population characteristics	n.a.
Recruitment	n.a.
Ethics oversight	n.a.

Note that full information on the approval of the study protocol must also be provided in the manuscript.

Field-specific reporting

Please select the one below that is the best fit for your research. If you are not sure, read the appropriate sections before making your selection.

[x] Life sciences [ ] Behavioural & social sciences [ ] Ecological, evolutionary & environmental sciences

For a reference copy of the document with all sections, see nature.com/documents/nr-reporting-summary-flat.pdf

Life sciences study design

All studies must disclose on these points even when the disclosure is negative.

Sample size	Sample size was chosen based on long-standing experience in the lab about the minimal number of samples needed to reliably detect biologically significant differences
Data exclusions	No data were excluded. Samples with technical failures were not considered data.
Replication	Experimental findings were reproduced in separate experiments as indicated in figure legends
Randomization	Experimental groups were not randomized
Blinding	Experiments were conducted in a non-blinded fashion

Reporting for specific materials, systems and methods

We require information from authors about some types of materials, experimental systems and methods used in many studies. Here, indicate whether each material, system or method listed is relevant to your study. If you are not sure if a list item applies to your research, read the appropriate section before selecting a response.

Materials & experimental systems

n/a	Involved in the study	
☐	☒	Antibodies
☐	☒	Eukaryotic cell lines
☒	☐	Palaeontology and archaeology
☐	☒	Animals and other organisms
☒	☐	Clinical data
☒	☐	Dual use research of concern
☒	☐	Plants

Methods

n/a	Involved in the study	
☒	☐	ChIP-seq
☐	☒	Flow cytometry
☒	☐	MRI-based neuroimaging

Antibodies

Antibodies used	All antibodies used are detailed in the manuscript's Methods section
Validation	Antibodies are validated in figures 1C, 3C and S2A,B or were from commercial vendors and validated as specified in the MSDS.

Eukaryotic cell lines

Policy information about cell lines and Sex and Gender in Research

Cell line source(s)	Cell lines and sources are specified in the manuscript's Methods section
Authentication	All cell lines were from renowned international repositories.
Mycoplasma contamination	All cell lines were tested for mycoplasma at regular intervals and were confirmed negative
Commonly misidentified lines (See ICLAC register)	None

Animals and other research organisms

Policy information about studies involving animals; ARRIVE guidelines recommended for reporting animal research, and Sex and Gender in Research

Laboratory animals	mus musculus
Wild animals	none
Reporting on sex	Animals of both genders were used to reduce the number of animals bred for research purposes.
Field-collected samples	none
Ethics oversight	Mouse experiments were carried out at the University of Basel in accordance with the Swiss law for animal protection and with authorization from the Cantonal veterinary office Basel-Stadt.

Note that full information on the approval of the study protocol must also be provided in the manuscript.

Plants

Seed stocks	n.a.
Novel plant genotypes	n.a.
Authentication	n.a.

Flow Cytometry

Plots

Confirm that:

- [x] The axis labels state the marker and fluorochrome used (e.g. CD4-FITC).
- [x] The axis scales are clearly visible. Include numbers along axes only for bottom left plot of group (a 'group' is an analysis of identical markers).
- [x] All plots are contour plots with outliers or pseudocolor plots.
- [x] A numerical value for number of cells or percentage (with statistics) is provided.

Methodology

Sample preparation	Described in detail in the manuscript's Methods section
Instrument	LSRFortessa (Becton Dickinson) and Aurora (Cytek) flow cytometers
Software	FlowJo software
Cell population abundance	Countless different populations are reported in the manuscript and their relative abundance reported at the appropriate place in the manuscript.
Gating strategy	Gating strategies are outlined in specifically designated supplementary figure panels

- [x] Tick this box to confirm that a figure exemplifying the gating strategy is provided in the Supplementary Information.
